# From Chemotaxonomy to Green Biocides: An Overview of New Studies on the Composition and Functional Properties of Some Plant Essential Oils

**DOI:** 10.3390/plants15101484

**Published:** 2026-05-13

**Authors:** Hazem S. Elshafie, Ippolito Camele

**Affiliations:** Department of Agricultural, Forestry, Food and Environmental Sciences, University of Basilicata, Via dell’Ateneo Lucano 10, 85100 Potenza, Italy; ippolito.camele@unibas.it

**Keywords:** essential oils, chemotaxonomy, green biocides, plant disease, pharmacological properties, sustainable agriculture

## Abstract

In recent years, the search for sustainable, bio-based alternatives to synthetic chemicals has intensified, positioning plant essential oils (EOs) at the forefront of applied phytochemical research. The following collection of ten articles from different geographical regions, published in *Plants* as part of the Special Issue “Plant Essential Oil with Biological Activity: 3rd Edition,” covers various aspects of recent scientific research on plant EOs, ranging from chemotaxonomy to green biocides, with particular emphasis on chemical composition and functional properties. Further attention is given to specific predominant single constituents and their bio-selectivity, modes of action, and innovative applications in the medical and pharmaceutical sectors, particularly against major diseases such as cancer and Alzheimer’s.

## 1. Introduction

In recent years, the demand for sustainable, bio-based substitutes for synthetic compounds has accelerated, establishing plant essential oils as central subjects in applied phytochemistry. Historically, EOs originate from ancient ethnobotanical practices, including Egyptian embalming (c. 3000 BCE) and traditional Ayurvedic and Greco-Roman medicine [1]. Recently, the application of plant essential oils has expanded into critical domains, including medicine, food technology, and plant protection, employing their antimicrobial, antioxidant, and biocidal properties [2,3].

Plant EOs exert their biological activity through a set of mechanisms that may target cellular integrity and metabolic function in microorganisms and pests [4,5]. The hydrophobic nature of EOs enables them to penetrate the cells and disrupt the cell membranes by increasing their permeability and causing leaks of some vital constituents such as proteins and nucleic acids [4]. This membrane disruption may be accompanied by interference with enzymatic systems involved in energy production including ATP synthesis and cell wall biosynthesis [4]. In addition, many EO constituents such as terpenes and phenolic compounds are also able to induce oxidative stress by generating reactive oxygen species, which can later damage the cellular components and harm microbial viability [6]. On the other hand, some EOs can inhibit microbial communication and biofilm formation by altering gene expression and interfering with quorum sensing process [7].

These different mode of action and possible mechanisms make EOs effective against a broad spectrum of harmful phyto- and human pathogens, whether bacteria or fungi. In addition, plant EOs prevent different pathogens from developing resistance towards the synthetic chemical and pharmaceutical agents. Moreover, the different modes of action explain their wide variety of uses in different fields including agriculture, food systems, and medicine.

## 2. Diversity, Bioactivity, and Applications of Plant EOs

The ten articles in this collection, published in *Plants*, explore the vast chemical diversity and biological potential of EOs derived from various botanical families, including Hypericaceae, Asteraceae, Rutaceae, Lamiaceae, and Apiaceae.

This Special Issue includes recent studies on plant EOs that span three main research areas: (i) chemical characterization and its link to bioactivity; (ii) agricultural applications, especially antifungal control; and (iii) biomedical and pharmacological development. The first group emphasizes chromatographic profiling to identify bioactive compounds, including selective butyrylcholinesterase (BuChE and AChE) inhibitors, as well as antimicrobial and antioxidant properties and chemical variability. The second group highlights agricultural uses, showing EO effectiveness against some serious fungal diseases and insect pests. The third group focuses on biomedical potential, including green biocide applications and notable cytotoxic activity against cancer cell lines. Overall, these studies demonstrate the multifunctional value of plant essential oils across diverse scientific fields.

### 2.1. Group A: Chemical Characterization and Bioactivity Screening

Calva et al. [8] investigated the EO from *Hypericum aciculare* (Hypericaceae) collected in southern Ecuador and chemically characterized its constituents using GC-FID and GC-MS analysis, revealing the presence of 53 constituents including hydrocarbon and aldehyde n-decanal as the predominant compounds. The study showed significant and selective inhibition against butyrylcholinesterase (BuChE) compared to acetylcholinesterase (AChE) enzymes. The authors concluded that *H. aciculare* EO is a promising candidate for further research into the mechanism of selective cholinesterase inhibition and potential natural therapeutic remedies for dementia.

Valarezo et al. [9] characterized the EO from *Ageratina dendroides* (Asteraceae), evaluated its biological activities and also determined its chemical composition through GC-MS and GC-FID. The obtained results demonstrated that this EO has notable antioxidant activity and moderate anticholinesterase activity. The findings highlight the importance of *A. dendroides* EO as a source of bioactive compounds with multiple promising pharmacological applications.

Zhigzhitzhapova et al. [10] investigated the EO of *Artemisia frigida* (Asteraceae), focusing on the stability and variability of its chemical profile, as EO components contribute to antioxidant activity, a key plant response to stress. Although EO composition responds to biotic interactions, general climatic changes have little effect on overall component ratios, and significant variations occur mainly under extreme conditions, such as high altitudes, exposed slopes, or human disturbance. The EO profile consists of a stable “core” of 24 compounds, likely under genetic control, and more variable components influenced by environmental and epigenetic factors. Its composition also changes across developmental stages and seasonal cycles.

Flores et al. [11], who provided a comprehensive analysis of the EO from the aerial parts of *Lasiocephalus ovatus* (Asteraceae), investigated its chemical composition, enantioselective profile, and preliminary biological activities. Using GC-FID/MS, the authors identified 71 compounds, predominantly sesquiterpene hydrocarbons and oxygenated sesquiterpenes. The main constituents included β-cyclogermacrene and spathulenol. The EO showed antibacterial activity against *Staphylococcus aureus* and *Escherichia coli*, as well as moderate antioxidant and anti-inflammatory effects, biological properties that are mainly attributed to its rich terpenoid profile, particularly oxygenated sesquiterpenes.

### 2.2. Group B: Antifungal and Agricultural Applications

Et-tazy et al. [2] investigated the in vitro antioxidant and antifungal activities of four EOs (*Rosmarinus officinalis*, *Myrtus communis*, *Origanum compactum*, and *Eugenia aromatica*) and their major chemical constituents against four post-harvest fungi affecting chickpea during storage (*Fusarium culmorum*, *Rhizopus oryzae*, *Penicillium italicum*, and *Aspergillus niger*). Results showed that all tested EOs exhibited varying levels of antifungal activity. In addition, they all displayed antioxidant properties, which may contribute to preserving seed quality by reducing oxidative stress during storage. This study highlights the potential of plant-derived EOs to act as dual-function bio-preservatives, combining antifungal and antioxidant effects. It also supports the use of natural products such as plant EOs as eco-friendly post-harvest protection agents in agricultural storage systems.

Moura et al. [3] evaluated the antifungal activity of citrus essential oil against sour rot in Tahiti acid lime caused by *Geotrichum citri-aurantii*. The results showed that citrus EO significantly reduced disease symptoms and inhibited fungal growth responsible for post-harvest decay. Its effectiveness is attributed to multiple mechanisms, including direct antifungal action and disruption of pathogen development processes. Treated fruits maintained better quality and exhibited an extended shelf life compared with untreated controls. Overall, the study demonstrates the potential of this essential oil as a natural alternative to synthetic fungicides and highlights its relevance for sustainable post-harvest citrus preservation and disease management.

Zhou et al. [12] investigated essential oils from patchouli, catnip, lavender, and mint (family Lamiaceae) for their bioactivity against *Thrips flavus* and their effects on crops and weeds. The oils exhibited strong insecticidal activity against the pest, as well as herbicidal effects on selected weed species. Additionally, they showed phytotoxic or growth-modulating effects on crop plants, indicating selective biological activity depending on plant type. These findings suggest that Lamiaceae essential oils have strong potential as eco-friendly agricultural agents for integrated pest and weed management, reducing dependence on synthetic agrochemicals while supporting more sustainable farming practices.

Elshafie et al. [4] undertook a broad investigation into the antifungal properties of plant essential oils against phytopathogenic fungi involved in plant diseases and post-harvest decay, reporting strong inhibitory effects across multiple fungal species, including reduced spore germination, impaired mycelial growth, and decreased pathogen viability. Proposed mechanisms of action include disruption of fungal cell membranes and interference with key metabolic processes. The authors emphasize the potential application of essential oils as natural fungicides in both pre- and post-harvest contexts, as their use could significantly reduce reliance on chemical fungicides, enhance crop protection, and contribute to environmentally sustainable plant disease management strategies.

### 2.3. Group C: Biomedical and Pharmacological Applications

In the review study carried out by Tanasă et al. [1], the authors highlighted the potential of EOs as natural, eco-friendly alternatives to synthetic biocides. They reported that EOs have significant antimicrobial, antibiofilm, and anti-quorum sensing activities. The authors focus on the role of EOs in preventing biofilms, which are responsible for the majority of microbial infections and the development of pathogen resistance. The study also addressed the mechanisms of action, such as disrupting cell membranes and regulating virulence factors, as well as the synergistic effect with antibiotics and the limitations of stability and selectivity.

For the first time, Vaglica et al. [13] investigated the chemical composition and cytotoxic properties of the EO from *Bifora testiculata*. GC-MS analysis revealed that it is rich in aliphatic aldehydes, with trans-2-dodecenal as the major component. When tested on human cancer cell lines (breast, melanoma, and colon), both the EO and trans-2-dodecenal exhibited remarkable cytotoxic activity, inducing apoptosis, with IC_50_ values ranging between 7.93 and 14.41 µg/mL for the EO and between 1.88 and 5.29 µg/mL for trans-2-dodecenal. These results indicated that the single compound was more potent than the crude EO. The use of the antioxidant N-acetyl-L-cysteine reversed this effect, indicating that the mechanism involves the generation of reactive oxygen species (ROS). The authors conclude that the EO’s activity is primarily due to its main aldehyde component, suggesting potential for further development as an anti-cancer agent.

## 3. Conclusions

In conclusion, the studies in this Special Issue highlight the chemical diversity of EOs from families such as Hypericaceae, Asteraceae, Rutaceae, Lamiaceae, and Apiaceae, demonstrating their strong biological activities, including antifungal, agricultural, and biomedical applications. Overall, this collection emphasizes the potential of EOs as versatile natural resources for sustainable agriculture and novel therapeutic development. The systematic screening approaches used also provide a reliable framework for identifying promising EO candidates for targeted applications in agriculture, the food industry, and medicine.

## Data Availability

Not applicable.
